# CyberKnife Treatment of Medically Intractable Trigeminal Neuralgia: A Comparison of Isocentric and Non-isocentric Treatment Planning Outcomes

**DOI:** 10.7759/cureus.79716

**Published:** 2025-02-26

**Authors:** Victor Tse, Jussi Sillanpaa, Ming Teng, Laura E Millender, Ann Y Minn, William Sheridan, Christopher McGuinness

**Affiliations:** 1 Neurosurgery, Kaiser Permanente, Redwood City, USA; 2 Radiation Oncology, Northwestern University Feinberg School of Medicine, Chicago, USA; 3 Radiation Oncology, Kaiser Permanente, South San Francisco, USA; 4 Neurological Surgery, Kaiser Permanente, Redwood City, USA

**Keywords:** cyberknife, facial numbness, outcome study, radiosurgery, trigeminal neuralgia

## Abstract

Background: Stereotactic radiosurgery (SRS) is commonly used to treat medically intractable trigeminal neuralgia. The target is either a point on the trigeminal nerve (isocentric treatment) or a finite length of the nerve (non-isocentric treatment). The superiority of one approach over the other is not well established. We compare our treatment outcomes for the two approaches in terms of pain relief and the most frequent side effect, facial numbness.

Methodology: A total of 83 consecutive patients (41 isocentric, 42 non-isocentric) were treated for trigeminal neuralgia with CyberKnife. Pain relief and facial numbness were scored using the Barrow Neurological Institute (BNI) scale, and the time to pain relief and facial numbness was recorded.

Results: There was no statistically significant difference in the probability of pain relief or the time to pain relief or facial numbness. Symptomatic numbness (numbness score >= III) was more prevalent in patients treated with the non-isocentric technique (isocentric 4, 9.8%; non-isocentric 10, 23.8%; *P *= 0.048).

Conclusions: Isocentric and non-isocentric techniques provide equivalent pain relief for trigeminal neuralgia. Symptomatic facial numbness is more prevalent when using the non-isocentric technique.

## Introduction

The efficacy of using stereotactic radiosurgery (SRS) to treat medically intractable trigeminal neuralgia (TN) is well established. When treating TN with GammaKnife (Elekta, Stockholm, Sweden), the isocentric technique is almost universally used. On the other hand, when treating with CyberKnife (Accuray, Sunnyvale, CA), the traditional approach was to contour a portion of the cisternal segment of the nerve and treat it using a non-isocentric technique. This was championed by the Stanford group and supported by Romanelli et al.’s [1] recent publication. They reported comparable results to the frame-based isocentric (targeting a point on the nerve) strategy [2,3]. In recent years, CyberKnife users have shifted to an isocentric strategy, while others continue to use the non-isocentric one. Both strategies aim to optimize the success of the treatment and mitigate facial numbness as a potential side effect. Symptomatic facial numbness is an undesirable but, to some extent, unavoidable side effect. This effect, known as *the Flickinger effect*, has been known for over 20 years, with multiple studies attempting to find the right balance between numbness and pain control. In this study, we compare the treatment outcome of these two strategies and estimate the probability of pain improvement and the potential in having facial numbness with each technique.

## Materials and methods

Patient population

We retrospectively reviewed our institutional database and accrued all patients who had TN treated by SRS from 2009-2017, according to a study protocol approved by the local institutional review board. Eighty-three consecutive patients without previous SRS for TN were included in this study. Their electronic records were reviewed and categorized into two groups: those treated with isocentric techniques and those treated with non-isocentric techniques. Of the 83 patients in the study, the first 42 patients were treated with the non-isocentric technique in irradiating a length of the nerve, while the next 41 patients were treated with the isocentric technique to a point on the nerve. The cohort treated with the non-isocentric technique had a longer follow-up time frame than the isocentric cohort. However, this difference did not affect the final Log-Rank analysis, as most events occurred within the overlapping time frames of both groups. The decision to treat these patients either with an isocentric or non-isocentric protocol was based on the neurosurgeon’s experience and preference. This selection was not biased by age or any other confounding factors (Table 1). The efficacy in pain control and the occurrence of facial numbness were analyzed. The study included 12 patients who had microvascular decompression before their SRS treatment (time interval of six months to seven years) and two patients who had prior glycerol injections (time interval of two to six years).

**Table 1:** Patient demographics, laterality, and affected nerve divisions. There was no difference in the age, sex, and laterality distribution between the isocentric and non-isocentric groups (*n* = 41 and 42, respectively, *P* = 0.743). In this study, trigeminal pain was more prevalent in females. It affected the right side of the face along the V2 distribution and was not different between the two groups (*n* = 41 and 42, respectively, *P* = 0.793).

Total patients	Isocentric, *n* (%)	Non-isocentric, *n* (%)
Number of patients	41	42
Median age (years)	73	72
Age range (years)	45-88	35-98
Sex		
Male	16 (39)	21 (50)
Female	25 (61)	21 (50)
Laterality		
Left	18 (43.9)	15 (35.7)
Right	23 (56.1)	27 (64.3)
Division		
1	3 (7.3)	0
1+2	3 (7.3)	13 (31.0)
2	18 (43.9)	15 (35.7)
2+3	3 (7.3)	9 (21.4)
3	10 (24.4)	3 (7.1)
1+2+3	4 (9.8)	2 (4.8)

Data tabulation

The outcome data were extracted from a prospectively established follow-up protocol for patients after treatment. We used the Barrow Neurological Institute (BNI) Scale to score pain and numbness in these studies (Pain: I, no pain, no medication; II, occasional pain, no medication; IIIa, no pain, on medication; IIIb, pain in control with medication; IV, some pain, not adequately controlled with medication; V, no pain relief). Facial numbness was scored according to the BNI scale as well (Facial numbness: I, no facial numbness; II, mild facial numbness; III: facial numbness, somewhat bothersome; IV: facial numbness very bothersome). Patients were followed up three months after the treatment, at 6, 8, 12, 18, and 24 months, and then yearly if there were no complications or issues. More frequent contact was undertaken if there were issues or concerns. Follow-up was done by phone or video visit and infrequently in person. Pain score, numbness, and medication were recorded.

Treatment protocol

**Figure 1:**
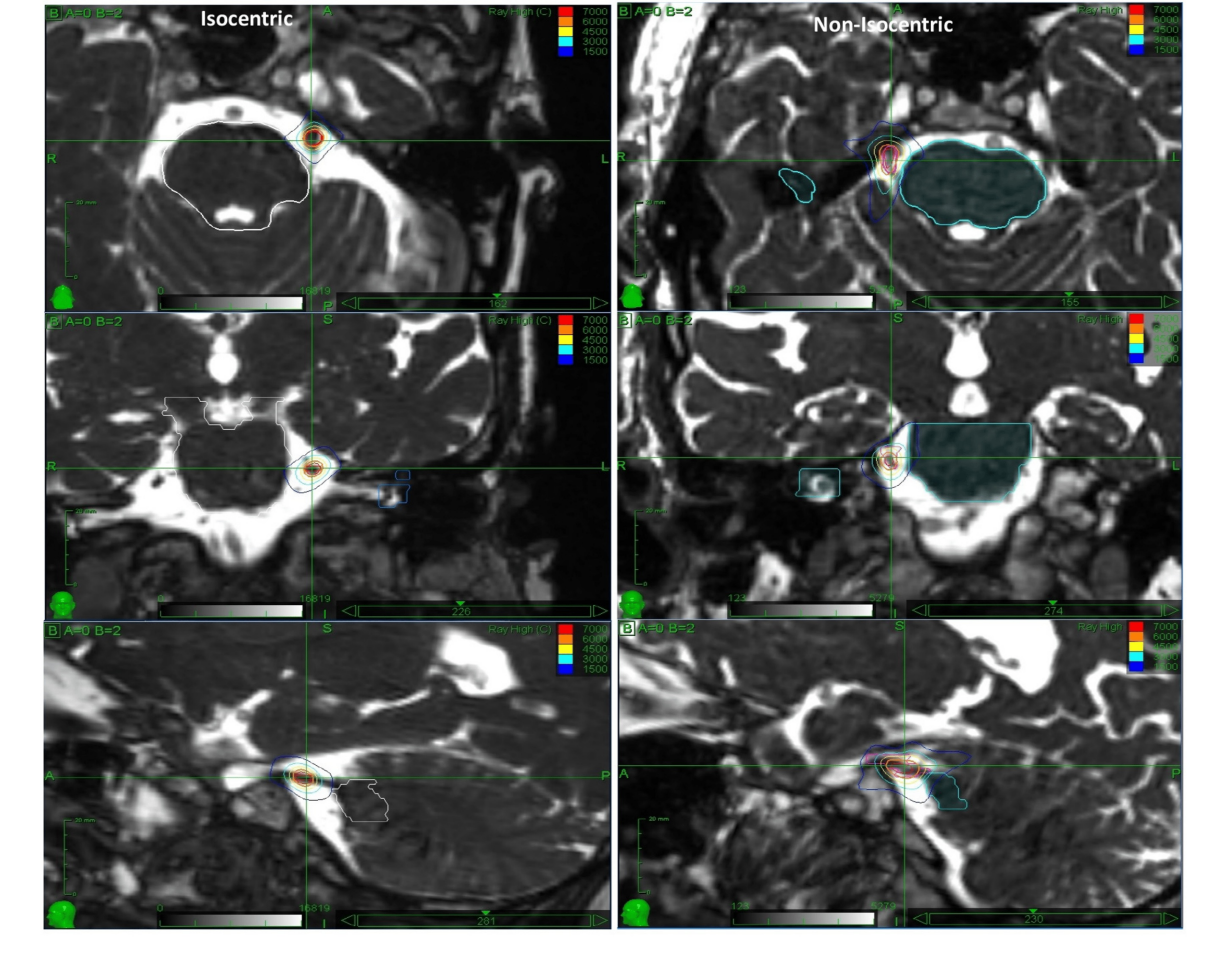
Treatment plan: A sample isocentric (left) and non-isocentric (right) radiation treatment plan. We routinely prevent beams from entering or exiting through the vestibular apparatus and conchae to reduce radiation exposure to these structures.

All the patients were imaged using the Fast Imaging Employing Steady-State Acquisition (FIESTA) sequence and were treated with CyberKnife using fixed cone collimators. The plans were delivered with the *trigem-specific* path at 650 mm source-to-axis distance (SAD) instead of the standard path at 800 mm SAD. This results in an effective 4 mm diameter treatment field. Figure 1 shows an example of isocentric and non-isocentric treatment plans. The location of the isocenter for the isocentric plans (typically 70-80 beams) was centered on the trigeminal nerve, 2-4 mm from the edge of the brainstem. The non-isocentric plans were optimized for a target that was approximately 6 mm in length along the nerve, starting approx. 4 mm from the brainstem and extending distally away from the brainstem. The isocentric plans were prescribed 85 Gy to 100% isodose, while the non-isocentric ones were prescribed 60 Gy to 80% isodose. The max dose to the brainstem was kept below 25 Gy, and the volume of the brainstem receiving 12 Gy was kept below 0.6 cc.

Statistical analysis

A description of patient characteristics (categorical, nominal, and ordinal) before and after treatment was tabulated and assigned a numerical value for dichotomous data analysis. We used the Wilcoxon signed-rank test for nonparametric analysis; other data were analyzed with analysis of variance (ANOVA) and Log-Rank regression. The calculations were performed using Matlab (MathWorks, Natick, MA) and Prism (Dotmatics, Boston, MA) software. The comparative review and summary result analysis was carried out by searching publications from major radiosurgery centers and selected publications that used BNI pain and/or BNI facial numbness score in their reporting of treatment outcome; the selected data were pooled, and the summary results for pain relief and symptomatic numbness were analyzed. The weighted average was estimated by weighting the outcome with the number of patients in the study, assuming the inter-study variance was the same. A case-control ratio (affected ratio) on affected numbness after treatment was calculated using the fixed effect method and presented as a Forest Analysis. The statistical significance and confidence level used in all analyses was 95%.

## Results

The median follow-up was 80 months (range 29-126 months; the non-isocentric patients had a longer follow-up). There were no significant demographical differences in sex or age distribution between the isocentric and the non-isocentric groups (Table 1). Trigeminal pain was more common in females and on the right side of the face, with the V2 distribution (33, 39.8%) being the most affected. Pain in all three divisions occurred only in 13 (7.2%) cases. The response rate to SRS remained high, even in patients with prior microvascular decompression or glycerol rhizotomy, with a success rate of approximately 70%.

For the non-isocentric treatments, the median prescription dose was 60 Gy (80% isodose line), and the median target volume of 0.069 cc. For isocentric treatments, the median dose was 85 Gy (100% isodose), and the median nerve volume was 0.033 cc receiving 60 Gy. At the last follow-up, 67 out of 83 patients (80.7%) had pain control (pain score <= IIIa), and 14 patients (16.8%) of patients with symptomatic numbness (numbness score >=3). The composite data for pain control and resultant facial numbness was tabulated in Table 2.

**Table 2:** Pain relief and facial numbness before and after treatment. Pain relief and facial numbness, assessed using the BNI scoring system, for both isocentric and non-isocentric treatments at the date of the last follow-up. Notably, patients with a pretreatment pain score of IV exhibited the most favorable responses.

	Isocentric, *n* (%)		Non-isocentric, *n* (%)	
	Pretreatment	Posttreatment	Pretreatment	Posttreatment
Pain score				
I	0 (0)	17 (41.5)	0 (0)	24 (57.1)
II	0 (0)	13 (31.7)	0 (0)	9 (21.4)
IIIa	0 (0)	4 (9.8)	0 (0)	0 (0)
IIIb	5 (12.1)	3 (7.3)	7 (16.6)	2 (4.8)
IV	33 (80.4)	4 (9.8)	33 (78.5)	4 (9.5)
V	3 (7.3)	0 (0)	2 (4.7)	3 (7.1)
Facial numbness				
I	0 (0)	26 (63)	0 (0)	17 (40.5)
II	0 (0)	11 (27)	0 (0)	15 (35.7)
III	0 (0)	2 (5)	0 (0)	7 (16.7)
IV	0 (0)	2 (5)	0 (0)	3 (7.1)

The overall findings indicated a statistically significant difference in symptomatic facial numbness (BNI scale III and above, *P *= 0.048). There was no difference in pain control between these two treatment plans (*P *= 0.44). It was noteworthy that patients with a pretreatment pain score of IV experienced the most favorable responses.

Pain control

**Figure 2:**
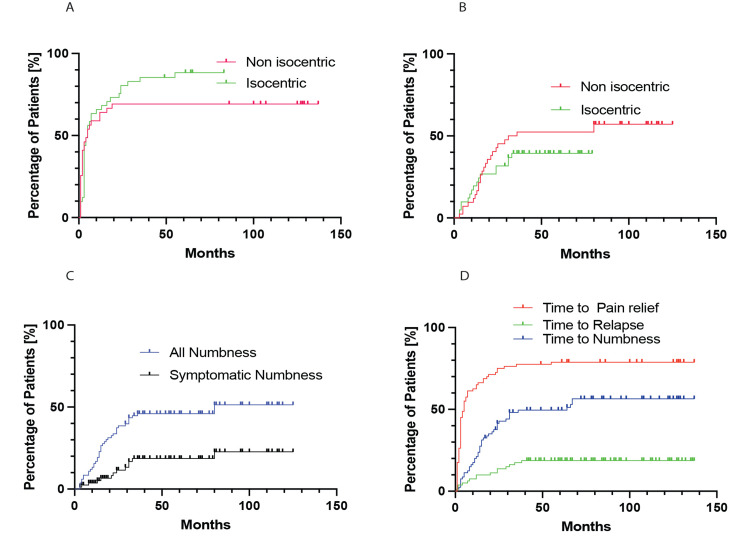
Temporal relationship of treatment efficacy and onset of facial numbness. Panel A illustrates the efficacy of CyberKnife treatment using isocentric and non-isocentric protocols. Panel B shows the onset of symptomatic numbness (BNI numbness score III or higher) in these protocols, while Panel C depicts overall numbness in the cohort. Panel D shows the timelines of pain relief, pain relapse, and numbness following SRS treatment. SRS, stereotactic radiosurgery

Initial response (pain score <= IIIa) at the first follow-up (three months) was 92.5%, decreasing over time to (89.9% at 12 months and 85.9% at 18 months) 80.5% at 24 months, after which pain control largely stabilized (Figures 2A, 2B, 2D). There was no significant difference in pain relief between the two strategies (34 patients in the isocentric group, 85.4%, and 33 patients in the non-isocentric group, 75.6%, *P *= 0.27). The median time to pain relief was three months (25 to 75 percentile, range 1.8-7.0 months). Notably, the latency in achieving pain relief was similar between the isocentric (mean 8.0 months, confidence interval [CI] 4.8-11.2 months) and non-isocentric groups (mean 7.2 months, CI 4.2-10.1 months). There was no difference in the efficacy between these two treatment plans as a function of time (Log-Rank *P *= 0.44) (Figure 2A). It was noteworthy that patients with a pretreatment pain score of IV were those with the most favorable responses (Table 2).

Facial numbness

Most patients experienced some degree of facial numbness. In our cohort, 14 patients (16.9%) reported symptomatic facial numbness (BNI score ≥ III) after SRS (Table 2). The relative risk of developing this was 3.08 (*P *= 0.001). Symptomatic numbness was more common in the non-isocentric group (10, 23.8%) compared to the isocentric group (4, 9.8%) (log-rank hazard ratio [HR] = 2.2, CI 0.73-6.42, *P *= 0.048). Facial numbness increased over time (Figures 2C, 2D), with a median onset of 14.5 months (25-75 percentile range: 8-24 months).

Facial numbness lagged behind pain relief (*P* = 0.0036, Fisher's exact test), with a median onset of 14.5 months (25-75 percentile range: 8-24 months), and stabilized after three years (Figure 2D). There was a correlation between successful treatment and the development of numbness (*r *= -0.27, *P *= 0.015). Prior treatments did not increase the risk of symptomatic numbness (*P *= 0.99). 

The likelihood of experiencing symptomatic numbness (BNI III-IV) following successful treatment (pain score reduced to ≤3a) is relatively high, estimated at 18%.

Cessation of pain medications

There was no correlation between pretreatment pain scores and the number of medications a patient took (*P *= 0.35). The median time for stopping or reducing medication was 20 months (range: 18.5-34.5 months). Despite being successfully treated (pain score IIIa), 4 (6.3%) patients continued taking medication prophylactically out of fear of recurrence. Overall, 41 patients out of 67 successfully treated patients stopped the medication, while 5 out of 67 patients reduced their medication.

Physics dynamics 

**Figure 3:**
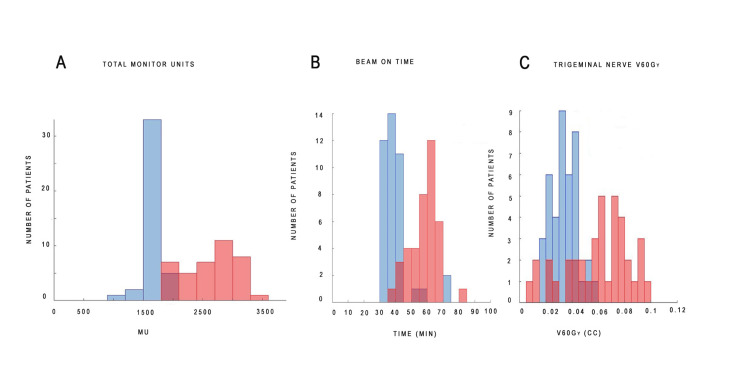
The plan metrics of isocentric and non-isocentric treatment paradigms. The plan metrics differed significantly between the two groups. The isocentric plan is shown in aqua pink, while the non-isocentric plan is shown in blue. The dark shade of color results from the overlap of blue and aqua pink. Total monitor units (Panel A) were 37% lower for isocentric treatments, and beam-on time (Panel B) was reduced by an average of 18 minutes compared to non-isocentric treatments. The dose to the trigeminal nerve (Panel C) was also lower in the isocentric group, with a mean V60 Gy of 0.034 cc compared to 0.059 cc in the non-isocentric group.

Isocentric treatments showed a 37% reduction in total monitor units and an 18-minute reduction in the beam-on time compared to non-isocentric treatments (Figure 3). The volume of the trigeminal nerve receiving 60 Gy was lower in isocentric treatments (mean V60 Gy: 0.034 cc) compared to non-isocentric treatments (0.059 cc). However, these parameters did not correlate with the treatment outcome.

## Discussion

Microvascular decompression separates the offending vessel(s) from the trigeminal nerve, eliminating neuronal ephaptic activities. This is an invasive yet non-destructive procedure, which provides almost immediate pain relief, allowing many patients to discontinue medications. Conversely, SRS is a non-invasive yet destructive procedure that uses irradiation to alleviate symptoms, revolutionizing trigeminal neuralgia management, as demonstrated in studies by Kondziolka et al., Young et al., and others [4-6]. With the advent of frameless stereotactic radiosurgery using linear accelerators (LINACs) came the concept of irradiating a longer segment of the nerve in the ambient cistern using a non-isocentric technique, aiming to minimize facial numbness and provide longer-lasting pain control [2-3]. Our retrospective study compared the outcomes of radiating a larger volume (a length of the nerve) and a single point on the nerve using CyberKnife, finding no statistically significant difference in long-term results between the two strategies. However, symptomatic facial numbness was found to be more likely for the non-isocentric treatments.

Our results are compatible with the existing literature. The rates of pain relief are 85.4% (isocentric) and 76.7% (non-isocentric). The overall success rate in pain control among selected, well-structured published studies ranged from 58% to 98% (estimated weighted average 83.3%) at 24 months and 58% to 81% (estimated weighted average 74.0%) at 36 months. We estimated the weighted average in the literature was 71.0% (58%-81%) and 75.2% (72%-76%) for isocentric and non-isocentric, respectively, at 36 months, a difference that is not statistically significant (*P *= 0.32).

**Figure 4:**
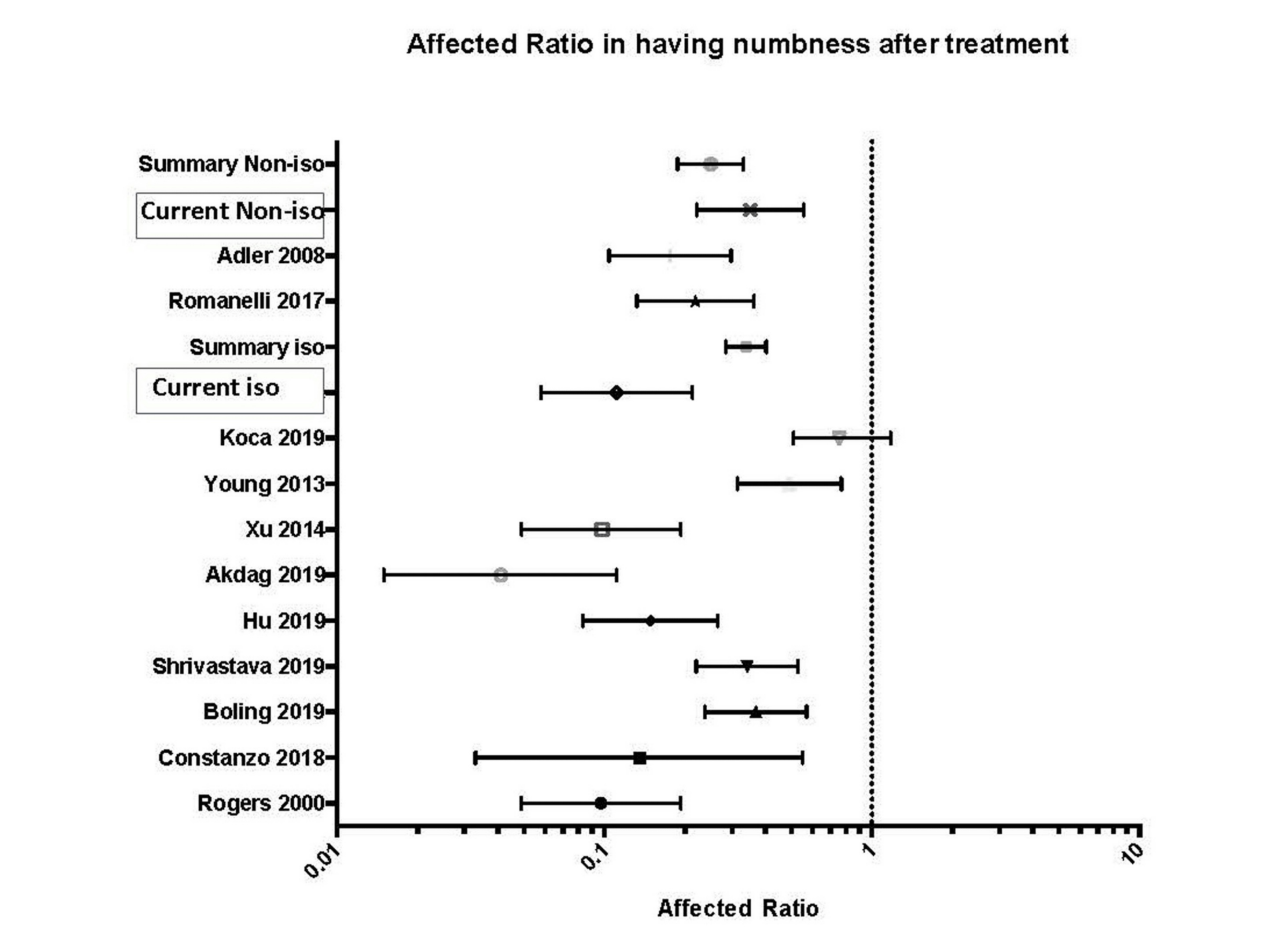
Forest plot showing the affected ratio of facial numbness after treatment. A graphical illustration of symptomatic numbness after treatment. This comparative review and summary analysis were conducted by searching publications from major radiosurgery centers, selecting studies that reported treatment outcomes using the BNI facial numbness score. Reported outcomes varied widely among studies using the isocentric technique. However, the overall affected ratio between the two techniques in this study - *Current Non-iso* and *Current Iso - *did not differ significantly, as shown in the summary effect. Data for affected ratios were extracted and recalculated from the following references, listed in descending order: [2], [1], [7], [8], [9], [10], [11], [12], [13], [14], and [6].

Most of the patients experienced some facial numbness after SRS. The number of patients in our study with symptomatic facial numbness (numbness>=III) was 16.8% (isocentric 9.8%, non-isocentric 23.8%). The amount of symptomatic numbness reported in the literature, estimated using the same method, was approximated to be 27.6% (26.5%-28.7%). The weighted symptomatic numbness we calculated for isocentric treatments was 24.2% (23.5%-24.8%), and for non-isocentric, 19.6% (17.2%-22.1%), a difference that is not statistically significant (*P *= 0.057; Figure 4). In our experience, a larger irradiation volume may increase the probability of symptomatic facial numbness.

Dmax and pain relief

It is generally believed that a maximum dose between 80 and 90 Gy to the nerve results in better long-term pain control, with most studies reporting an 85% to 95% response rate within the first year [7,9,12,13,15], which inevitably declines over time. The weighted average for having pain relief after 36 months is approximately 74%. Administrating doses at the upper end of this range resulted in a more robust response and a shorter latency between treatment and pain relief [16]. The latter was one to three months in most studies. The trade-off in using a higher dose is an increased risk of symptomatic facial numbness [8,13,17].

Irradiation volume and its effect on pain relief

Kondziolka et al. showed that a higher dose in a smaller volume yielded longer-lasting results. In a seminal study, Flickinger et al. [18] showed that there was no difference in pain relief when using larger volumes (one vs. two isocenters). They extrapolated this result to conclude that irradiating a longer segment or larger volume of the nerve had no impact on pain relief. Some investigators advocate adjusting the length and volume of the target to optimize treatment efficacy, while others opt for selecting the anatomical location of the target. The optimal nerve length or volume to be irradiated remains controversial. This is partly due to variations in the length of the cisternal segment of the nerve (5-14 mm) [19], its angle of emergence from the brainstem (24-38 degrees) [17], and the angle of its ascending course toward Meckel’s cave (150-170 degrees) [5], all of which influence the effective amount of the nerve being irradiated at the prescribed dose. Régis et al. [20] advocated placing the target 7.6 mm from the emergence point. 

Recent studies by Shrivastava et al. [12] and Mousavi et al. [19] examined the integral dose (ID), defined as the product of dose and volume. Both studies showed that ID correlated well with treatment outcomes. The optimal ID dose was in the range of 1.4-2.7 mJ. This was translated to a median Dmax of 81.1 Gy (80.7-81.5) and an irradiated volume of 29.8 mm³ (28.5-31.0); these parameters were compatible with those used in our study. Kondziolka et al. [4] concluded a higher dose to a smaller volume of the nerve trended toward longer-lasting pain control.

Irradiation volume and facial numbness

Rashid et al. [17] demonstrated that a larger target volume (median volume of 0.018 mm³) receiving 70 Gy tended to result in better pain relief with acceptable numbness. Adler et al. [2] championed the idea of irradiating a segment of the nerve. In his study, a 6 mm length of the nerve receiving 74.5 Gy yielded a reasonable outcome without increasing the risk of numbness (72% pain relief and 15% numbness). Constanzo et al. [14] and Flickinger et al. [18] reported a direct correlation between target length/volume and numbness. Mousavi also found a higher ID could potentially increase facial numbness [19]. The non-isocentric treatment volume used by Romanelli et al. [1] was smaller (30 mm³) than the V60 Gy used in this study, which explains the negligible overall numbness rate in that study. Collectively, our results support the notion that a larger irradiation volume can increase the chance of symptomatic facial numbness.

The temporal relationship between pain relief and numbness

Intuitively, one might expect numbness to occur before pain relief after SRS. However, our study confirms that pain relief precedes the onset of symptomatic numbness. In our study, the median onset of numbness after treatment was 14.5 months (8-24 months). Similar results were reported by Régis et al. [20], Romanelli [1], and Constanzo [14]. Any onset of numbness more than three to four years after treatment is unusual. Unfortunately, numbness is permanent in many of the patients [10, 11]. For a patient who is successfully treated, we estimated that the conditional probability of developing symptomatic numbness later is 18%. Fortunately, quality-of-life analyses by Régis et al. [20] and Young et al. [8] confirmed most patients are willing to trade pain for numbness.

Treatment failure

Treatment failure happened relatively early after treatment. In our study and others, despite early pain relief, relapse commonly occurred one to two years after treatment [1,9,15]. In a study by Rogers et al. [6], only 41% of successfully treated patients were able to discontinue medications entirely, while another 30% reduced their intake by at least 50%. The remaining patients experienced only minor or no adjustments to their dosage. We found that 6.3% of our successfully treated patients (pain score IIIa) remained on medications. Some patients were unable or unwilling to discontinue medications even after successful treatment [21], likely due to pain catastrophizing or pain-related anxiety.

This is a retrospective study with limited follow-up duration and is statistically underpowered. Despite these limitations, we demonstrated that the difference in pain relief between isocentric and non-isocentric planning for TN treatment is minimal. We also found that isocentric treatment causes less symptomatic facial numbness (*P *= 0.048). Importantly, the beam-on time is shorter for isocentric treatments, providing benefits for patient comfort. We believe isocentric treatments are more appropriate for patients who are fidgety or have difficulty tolerating lying still.

## Conclusions

Although the exact pathophysiology of TN and the mechanism by which SRS alleviates its symptoms are not completely understood, SRS is a good treatment option for this condition. We achieved an overall successful rate of 80.5% and an unwanted, symptomatic facial numbness rate of 16.9%, which agrees with other SRS studies in the literature. We also found that symptomatic (BNI score >= III) facial numbness was more likely with non-isocentric treatments (*P *= 0.048). Isocentric treatments have shorter beam-on times, improving patient comfort. We believe isocentric treatments are more suitable for patients who have difficulty lying still.

Patients with BNI pain class IIIa after treatment should be given assurance that a long-lasting result is expected. The probability of experiencing a treatment failure after four to five years is low. They may want to discontinue their medication at that time to reduce therapeutic risk. Numbness is a predictor of long-lasting pain relief, and any new onset of numbness more than three to four years after treatment is uncommon.
